# Canalizing cell fate by transcriptional repression

**DOI:** 10.1038/s44320-024-00014-z

**Published:** 2024-02-01

**Authors:** Bryce Lim, Katrin Domsch, Moritz Mall, Ingrid Lohmann

**Affiliations:** 1 Cell Fate Engineering and Disease Modeling Group, German Cancer Research Center (DKFZ) and DKFZ-ZMBH Alliance, 69120 Heidelberg, Germany; 2 HITBR Hector Institute for Translational Brain Research gGmbH, 69120 Heidelberg, Germany; 3 Central Institute of Mental Health, Medical Faculty Mannheim, Heidelberg University, 68159 Mannheim, Germany; 4 Heidelberg University, Centre for Organismal Studies (COS) Heidelberg, Department of Developmental Biology and Cell Networks - Cluster of Excellence, Heidelberg, Germany

**Keywords:** Alternative Fate Repression, Cell Identity, Cell Fate Plasticity, Epigenetic Silencing, Transcriptional Repressor, Chromatin, Transcription & Genomics, Development

## Abstract

Precision in the establishment and maintenance of cellular identities is crucial for the development of multicellular organisms and requires tight regulation of gene expression. While extensive research has focused on understanding cell type-specific gene activation, the complex mechanisms underlying the transcriptional repression of alternative fates are not fully understood. Here, we provide an overview of the repressive mechanisms involved in cell fate regulation. We discuss the molecular machinery responsible for suppressing alternative fates and highlight the crucial role of sequence-specific transcription factors (TFs) in this process. Depletion of these TFs can result in unwanted gene expression and increased cellular plasticity. We suggest that these TFs recruit cell type-specific repressive complexes to their cis-regulatory elements, enabling them to modulate chromatin accessibility in a context-dependent manner. This modulation effectively suppresses master regulators of alternative fate programs and their downstream targets. The modularity and dynamic behavior of these repressive complexes enables a limited number of repressors to canalize and maintain major and minor cell fate decisions at different stages of development.

## Abstract (web summary)

This Review discusses the key role of transcriptional repression in cell fate regulation. It provides an overview of epigenetic mechanisms and transcription factors suppressing undesired gene expression or alternative cell fates and discusses the implications for cellular reprogramming.

## Graphical abstract

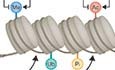

## Introduction

**Figure 1:**
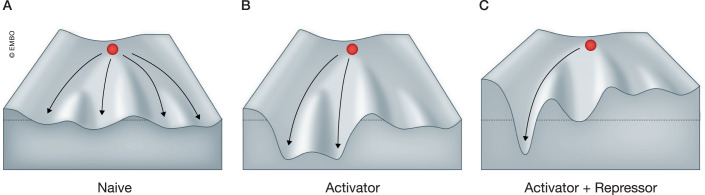
Repressors and activators jointly shape Waddington’s epigenetic landscape to canalize cell fate. (**A**) The developmental trajectories of stem and progenitor cells are initially unrestricted, offering multiple potential routes. (**B**) Cell fate-inducing transcriptional activators, like master regulators, guide specific differentiation trajectories. Using the metaphor of Waddington’s landscape, a master regulator can carve out valleys leading toward one or multiple cell identities. (**C**) Transcriptional repressors that silence alternative gene regulatory programs help stabilize specific cell identity trajectories. Such repressors can be depicted as raising the height of the mountains between different fates. In tandem with activators, these repressors can enable complete canalization of cell fate, enhancing the fidelity of cell fate determination during development and preventing plasticity later in life.

Stem and progenitor cells have the potential to differentiate into every cell type within multicellular organisms along distinct trajectories. This process ensures the faithful generation of diverse and specialized cell types, a concept nicely illustrated by Waddington’s epigenetic landscape model (Fig. 1). The establishment of precise gene expression patterns is crucial in guiding cell fate determination and maintenance, ensuring proper development and function of diverse cell types. Six decades of research since Jacob and Monod’s discovery of dedicated transcriptional repression mechanisms in bacteria (reviewed in (Gann, 2010)) have emphasized the critical role of repression. The balanced interplay between activation and repression sculpts Waddington’s landscape (Fig. 1), orchestrating cell-type-specific gene expression programs and thereby regulating cell identities during development (Gray and Levine, 1996; Hirabayashi and Gotoh, 2010; Lunyak and Rosenfeld, 2008; Young, 2011). Disruptions in finely tuned gene expression programs may increase cellular plasticity, a mechanism that has been suggested to impair function even in mature cells, possibly contributing to cognitive decline, aging, and cancer (reviewed in (Denslow and Wade, 2007; Hanahan, 2022; Pal and Tyler, 2016; Sweatt, 2010).

The establishment of cell type-specific gene expression programs requires precise regulation of target genes. This regulation is facilitated by interactions between specific transcription factors (TFs) and their DNA-binding sequences within gene regulatory elements, such as proximal promoters and distal gene regulatory elements. In general, TFs can induce transcription (activators) or silence gene expression (repressors), and often have dual roles depending on the target genes and developmental context. In humans, over 1600 TFs are thought to exist, with a broad range of DNA-binding domain (DBD) types: Cys2His2-type (C2H2) zinc finger and homeodomain (HD) TFs comprise over half of known or predicted TFs, and most of the remainder are composed of basic helix-loop helix (bHLH), basic-leucine zipper (bZIP), Forkhead, nuclear hormone receptor, high mobility group (HMG)/Sox, or erythroblast transformation specific (ETS) TFs (Lambert et al, 2018). It is difficult to classify TFs as definitively repressive or activating. Approximately 400 different non-DBD effector domains have been identified across the human TF repertoire, each with different mechanisms of regulating gene expression or protein function. However, some effector domain types are more abundant than others and have more defined functions; for instance, the repressive KRAB domain is present in roughly 350 human TFs (Lambert et al, 2018).

**Figure 2:**
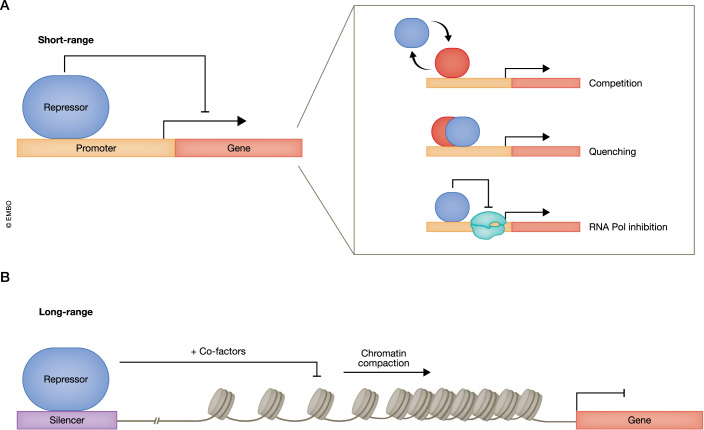
Transcriptional repression occurs at both short and long ranges. (**A**) Repressors (blue) can directly downregulate transcription at promoter-bound genes through competition with activators (red), quenching of activators, or inhibition of RNA polymerase (green). (**B**) Repressors can exert their influence at longer ranges by binding to silencers and recruitment of cofactors and complexes, such as Polycomb Group (PcG) proteins that can trigger the formation of inactive heterochromatin domains resulting in the downregulation of distal genes.

In this review, we will focus on transcriptional repression that we define as the active process by which transcription of specific genes or gene programs is either decreased or continuously switched off to regulate the state or identity of a cell. Based on the examples presented in this review, we consider repression to be an active process, and not just the absence of activation, which is critically important to regulate cell fate. We will review the two main mechanisms and associated machinery known to silence target genes via short-range and long-range repression (Fig. 2). Short-range repression can occur by TF binding directly to a gene promoter, typically defined as being within 100–1000 bp from the transcriptional start site (TSS). Several mechanisms can contribute to this process, including activator competition, where the repressor competes with an activator protein for binding to the gene promoter (Gray and Levine, 1996; Liu et al, 2021). In addition, classic models describe that short-range repression can occur through activator quenching, whereby repressors bind to regulatory regions to interact locally with nearby activators and or directly with RNA polymerase to decrease transcription (Fig. 2A) (Gray and Levine, 1996; Thiel et al, 2004).

Long-range repression occurs by TF binding to distal elements known as silencers, which can then regulate transcription at linked promoters several mega-bases away by chromatin looping (Brand et al, 1985; Friedman et al, 2021). In this way, these elements are analogous to enhancers but act to downregulate instead of activate gene expression. It is important to distinguish silencers from insulators, which block enhancer activity. >50,000 enhancers are thought to exist in Drosophila (Pfeiffer et al, 2008), and up to a million in humans (Heintzman et al, 2009; Thurman et al, 2012). It is not clear if a similar number exists for silencers (Doni Jayavelu et al, 2020). However, it is known that many enhancer elements also act as silencers in different cellular contexts, or vice versa. This dual activity may be present in ~10% of enhancers (Gisselbrecht et al, 2020; Huang and Ovcharenko, 2022). There have been attempts to characterize and predict silencer elements, but no consensus exists on a definition for, or common features of, silencers, except that they should repress gene expression, which is one of the problems when testing predictions. Both short- and long-range repression is often mediated by epigenetic modifications. These include DNA methylation and histone modifications that can lead to the generation of heterochromatin (Bannister and Kouzarides, 2011; Liang et al, 2017; Watson et al, 2012). These modifications can silence gene expression by rendering DNA less accessible to most TFs, with the notable exception of pioneer factors (Barral and Zaret, 2023), as discussed below (section: “Enhanced reprogramming by specific repression”) and RNA polymerase, to decrease gene transcription (Gray and Levine, 1996; Payankaulam et al, 2010). They can have long-lasting effects and may be heritable, playing a critical role in development and differentiation (Fig. 2B).

Here, we provide an overview of cell fate regulation by repression, discussing general epigenetic mechanisms and specific TFs known to suppress unwanted gene expression or alternative cell fates. Finally, we explore strategies to identify such repressors, and examine the potential utility of repressors in cellular reprogramming.

## Epigenetic alternative fate repression: long-term silencing by covalent modifications

**Figure 3:**
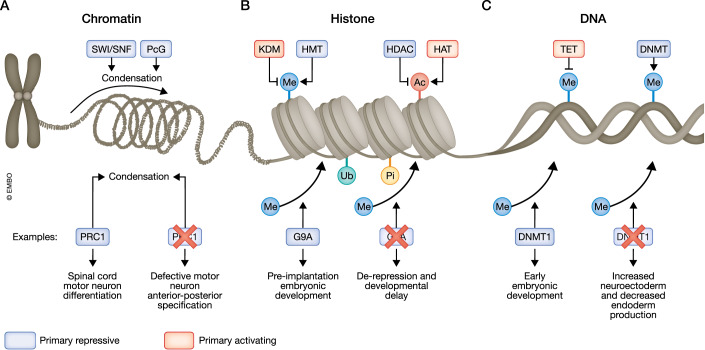
Repressive epigenetic machinery across different molecular scales. Distinct enzymes are responsible for repression at different scales, making modifications to higher-order chromatin structure, histones, or DNA. Machinery at all three levels have been associated with repression-associated cell identity regulation as shown by indicated examples. (**A**) Chromatin remodelers include complexes that contain Polycomb Group (PcG) proteins, and the repressive SWItch/Sucrose Non-Fermentable (SWI/SNF) complex and have been linked to regulation of cell fate by repression. For example, loss of Polycomb repressive complex 1 (PRC1) in motor neuron progenitors leads to errors in repression of various fate determinants, particularly Hox TFs, during motor neuron specification in mice (Sawai et al, 2022). (**B**) A broad range of histone modifications exist, and these modifications are added or removed from histone tails by specialized enzymes including lysine demethylases (KDMs) and histone methyltransferases (HMTs), which modulate methylation (ME), and histone deacetylases (HDACs) and histone acetyltransferases (HATs), which catalyze acetylation (Ac). Many other modifications and associated enzymes exist including, but not limited to, ubiquitination (Ub) and phosphorylation (Pi). Many of these enzymes regulate development; for instance, loss of the HMT, G9A, leads to derepression of 4-cell stage-related genes in 8-cell mouse embryos. This led to delayed development, improper lineage segregation, and impaired lineage stability (Zylicz et al, 2018). (**C**) Finally, DNA modifiers such as ten-eleven translocation (TET) enzymes and DNA methyltransferases (DNMTs) can add or remove DNA modifications, particularly methyl groups (Me), that can contribute to gene silencing. For example, deletion of DNMT1 during mouse embryonic development causes increased differentiation toward the neuroectodermal lineage and decreased endodermal lineage commitment (Grosswendt et al, 2020).

Intricate layers of repressive epigenetic modifications at the DNA, histone, and higher-order chromatin levels suppress alternative gene regulatory programs to maintain cell fate decisions. Crucial to this repression during cell fate determination are general remodeling complexes like SWI/SNF and Polycomb (PcG) complexes (Fig. 3A). Histone modifying complexes containing histone deacetylases (HDACs), such as NuRD, CoREST, and Sin3 complexes, or complexes containing histone methyltransferases, like G9/GLP, SUV39H1/2, and SETDB1, also play significant roles (Fig. 3B). Finally, DNA methyltransferases (DNMTs) mediate the repression of non-lineage genes during development (Fig. 3C). The roles of these complexes and their associated proteins in transcriptional repression have been extensively discussed in comprehensive reviews (Blackledge and Klose, 2021; Bogdanović and Lister, 2017; Brand et al, 2019; Laugesen and Helin, 2014; Padeken et al, 2022). Although outside the scope of this review, it is worth mentioning that many of these enzymes have non-histone targets in the nucleus and cytoplasm (Li et al, 2020; Rai et al, 2007; Shen et al, 2018), which could indirectly affect cell fate. Below, we highlight recent studies that provide additional insights into the functions of epigenetic regulators in repressing alternative cell fates.

### Epigenetic repression in stem cells and during differentiation

Polycomb proteins form two primary complexes: Polycomb repressive complex 1 (PRC1) and Polycomb repressive complex 2 (PRC2). These collaborate to silence target genes by modifying chromatin structure. PRC2 is responsible for the initial recruitment to target genes and the deposition of the repressive histone mark H3K27me3, catalyzed by the histone methyltransferase EZH2. This modification acts as a signal for PRC1 to bind to the chromatin and reinforce gene silencing. PRC1 comprises various subunits, including RING finger domain-containing proteins (RING1A/B) and chromodomain proteins (CBX2/4/6/7/8), which promote chromatin compaction and impede transcriptional initiation (Brand et al, 2019). In addition to their chromatin modification activity, it should be noted that these enzymes also have other targets. For instance, RING1 also acts independently of PRC1 to ubiquitinate p53 and target it for degradation (Shen et al, 2018).

While PRC1 and PRC2 have been extensively studied in embryonic stem cells (Laugesen and Helin, 2014), their roles in adult stem cells are less comprehensively understood. Recent studies on adult intestinal stem cells (ISCs) and hair follicle stem cells (HFSCs) have demonstrated that PRC1 is crucial in repressing non-lineage-specific TFs to maintain cell identity in a context-dependent manner (Pivetti et al, 2019), but there are some important differences. Depletion of PRC1 in HFSCs led to the activation of an epidermis-specific gene expression program, thereby impairing hair regeneration. In contrast, PRC1 reduction in ISCs, despite causing the de-repression of non-lineage TFs, did not initiate an alternative transcriptional program, but interfered with the ISC-specific program, resulting in a similar phenotype: stem cell depletion (Pivetti et al, 2019). Intriguingly, this study showed that while H3K27me3 marks were largely sufficient to prevent TF expression only a few of the PcG targets contributed to the phenotype. This suggests that PRC1 has a broad function in sustaining the identity of adult stem cells, leading to a common phenotype that relies however on specific mechanisms tailored to each cell type. The effects of PRC1/2 are potentially influenced by the specific epigenetic landscape and TFs already expressed in a given cell type, rendering each cell susceptible to a specific range of cell fate or program changes.

The role of PRC1 and PRC2 was also examined during the differentiation of spinal cord motor neurons (MNs) by knocking down the PRC2 subunits Ezh and Eed, as well as the PRC1 subunit RING1B, in mouse MN progenitor cells (Sawai et al, 2022). Intriguingly, this study revealed that genetic reduction of PRC2 components did not affect MN development, but PRC1 depletion caused defects in the diversification of MN subtypes due to the lack of repression of various fate determinants, particularly Hox TFs (Fig. 3A). Nonetheless, the loss of PRC1 did not result in the acquisition of features associated with alternative neuronal classes, as the MNs continued to express general motoneuronal markers (Sawai et al, 2022). Hence, PRC1 appears to have a critical role in restricting the expression of cell-fate determinants along the rostrocaudal axis, defined by the Hox code, while the absence of PRC2 can be compensated for. How is this possible? One explanation is that in PRC2 mutant cells, residual H3K27me3 is inherited during cell division, which is sufficient to attract canonical PRC1 and sustain the repression of target genes in postmitotic neurons. This aligns with the understanding that PRC1 anchoring to the chromatin is independent of high H3K27me3 levels but is instead driven by the assembly of phase-separated sub-nuclear structures through the polymerization of Polyhomeotic-like and/or Cbx proteins into Polycomb bodies attracted by residual H3K27me3 levels (Plys et al, 2019).

In addition to the distinct functions of different PRC complexes in development, even the same PcG components can govern opposing lineage decisions in a stage-dependent manner, as observed during gliogenesis. In the mouse neocortex, neural progenitor cells (NPCs) progress through a neurogenic phase where they generate neurons, followed by a gliogenic phase where they produce glial cells. PRC2 orchestrates both phases by inhibiting early gliogenesis during the neurogenic phase and limiting neuronal differentiation during the transition to the gliogenic phase, thereby promoting astrogenesis. CHD4, a chromodomain helicase, has been identified as a crucial interaction partner of the PRC2 component EZH2, specifically required for the PcG-mediated repression of the critical glial marker gene *GFAP* during the neurogenic phase. As the transition from the neurogenic to astrogenic phase occurs, Polycomb proteins begin to repress the expression of the pro-neuronal gene *Ngn1*, thereby limiting the neurogenic potential of NPCs and permitting gliogenesis (Sparmann et al, 2013). These findings highlight the dynamic nature of PcG protein interactions and their association with “unconventional” cofactors like CHD4, a core component of the repressive NuRD complex at different developmental stages. This adaptability enables the same repressive core machinery to govern distinct cell fate transitions by repressing very distinct and few target genes in a H3K27me3-mediated manner using stage-specific cofactors.

Similarly, gliogenesis in *Drosophila* relies on Glial cells missing (Gcm), a TF belonging to the GCM domain superfamily, which plays a pivotal role in determining whether multipotent neuronal progenitors adopt a glial or neuronal fate. The fate decision is controlled by the transient expression of Gcm, and this temporal regulation of Gcm expression is under the control of PcG proteins. Specifically, the PRC1 component Polycomb (Pc), a paralog of vertebrate CBX2/4/6/7/8, directly represses *gcm* transcription by competing with Gcm at its autoregulatory control region (Popkova et al, 2012). This highlights the specificity of repressive chromatin remodelers for their target genes, which allows them to modulate the expression of a single transiently expressed cell fate determinant, critical for the acquisition of a specific cell identity.

### Epigenetic silencing in sex determination

The precise control of opposing cell fate decisions is crucial for sex determination in gonads, which comprise somatic support cells and germ cells. In mammals, both XX and XY fetal gonads have the potential to differentiate into either testes or ovaries. Testicular fate determination is dependent on the proper function of the PRC1 subunit CBX2 in somatic support cells. Loss of *CBX2* in these cells results in hypoplastic ovaries instead of testis. CBX2 silences ovarian-determining genes, preventing these genes from indirectly suppressing testis development, which is contrary to previous assumptions of CBX2 acting as an activator of the testis-determining gene Sry (Garcia-Moreno et al, 2019). Mechanistically, many genes of the Wnt pathway are repressed by PcG-dependent H3K27me3-mediated repression. Specifically, *Lef1*, an ovary-promoting gene downstream of Wnt, has been identified as a target of CBX2 binding (Garcia-Moreno et al, 2019). This study also showed that bivalent epigenetic signatures persist at a substantial number of sex-determining genes controlling the alternative pathway even after sex determination has occurred, possibly preserving their dual developmental potential. Conversely, the presence of bivalent histone marks on genes associated with alternative fates may serve as a prognostic indicator for the susceptibility of these genes to induce undesired fate transitions upon de-repression in pathological conditions.

Proper gonad function requires concordance between the sexes of the somatic and germline lineages. How is proper sex determination ensured in the germline? Unlike the role of PcG in the testis, in *Drosophila* the identity of female germ cells is ensured by the epigenetic writer SETDB1. This methyltransferase tri-methylates Lysine 9 on histone H3 at critical genes involved in spermatogenesis, thereby silencing male genes and safeguarding female germ cell identity (Smolko et al, 2018). Recruitment of SETDB1 to male genes relies on the *Drosophila* master sex-switch factor Sex lethal (Sxl), a female-specific RNA binding protein. Remarkably, SETDB1-dependent H3K9me3 deposition is highly localized, does not spread to adjacent loci, and can even be restricted to transcriptional start sites (Smolko et al, 2018). Loss of SETDB1 in the female germline leads to a block in germ cell differentiation, resulting in a tumor phenotype (Smolko et al, 2018). Like in the case of ISCs and HFSCs (Pivetti et al, 2019), this highlights the important role that epigenetic repressor molecules play in adult stem cell control. However, while in the case of ISCs and HFSCs activation of non-lineage TFs resulted in stem cell depletion, in female germ cells a different phenotype is observed, namely the accumulation of germ cells unable to differentiate.

### Epigenetic control of alternative fate repression in early development

Epigenetic regulators play a crucial role in early mouse development. For example, the absence of the H3K9 histone methyltransferase G9A, which accumulates at the 4- to 8-cell stage, led to higher expression of 4-cell stage genes and TFs, including Tead4, at the 8-cell stage, causing developmental delay and defects in inner cell mass lineage specification (Fig. 3b) (Zylicz et al, 2018). A study using single-cell RNA-sequencing (scRNA-seq) examined mouse embryonic development from pluripotent epiblast stage to early organogenesis, focusing on the effects of depleting several key epigenetic regulators, including G9A, the DNA methyltransferases DNMT1-3, and core components of PRC1 and PRC2 (Grosswendt et al, 2020). PRC-associated mutants caused the most consistent defects in terms of transcription and morphology during gastrulation in this model. PRC1 and PRC2 cooperate to ensure ectodermal differentiation and induction of neural ectoderm by repression of posterior lineages including extraembryonic mesoderm. Conversely, deletion of DNMT1 during mouse embryonic development causes increased differentiation toward the neuroectodermal lineage and decreased endodermal lineage commitment (Fig. 3c). However, PRC2 mutants showed more severe defects, including excessive primordial germ cell production, indicating the dominance of this complex in early lineage restriction (Grosswendt et al, 2020). Whether the altered extraembryonic tissues caused at least some of the differentiation defects, e.g., via changes in morphogen gradients, remains to be investigated.

Exit from pluripotency coincides with the establishment of an extensive repressive epigenetic environment, which has been revealed by single-cell profiling of the DNA methylome, nucleosome, and transcriptome of early mouse development at the transition stage between exit of pluripotency to germ layer specification (Argelaguet et al, 2019). This is followed by the emergence of lineage-specific epigenetic patterns during gastrulation. The study suggests that the ectodermal lineage is established in the early epiblast, indicating that it is the default state. In this model, endoderm and mesoderm are actively diverted from the default ectodermal pathway, in contrast to some findings of Grosswendt and colleagues (Grosswendt et al, 2020). However, both studies indicate a need for active transcriptional repressors of alternative fates to ensure proper commitment and specification of these germ layers during early mouse development.

In summary, epigenetic regulators, despite their universal expression, function in a highly developmental time-specific manner to suppress alternative cell fates. This ensures the initiation and robust maintenance of cell fate decisions. In most cases, specificity in the recruitment of these regulators or complexes to particular genomic sites is achieved through dynamic interactions with a variety of cofactors, in particular sequence-specific TFs, which we will discuss in the following section.

## Cell and tissue specificity of alternative fate repression: the role of TFs

Transcriptional repression, which enables targeted silencing of specific genes, involves the binding of repressors to specific DNA sequences. Mechanistically, repressors employ two main strategies (Fig. 2). First, they can indirectly impede gene expression by either blocking the binding of activators or by regulating the activity of activators and RNA polymerases. Second, they can work in conjunction with ubiquitous, DNA sequence-unspecific epigenetic machinery to modify nearby DNA and chromatin, thereby altering target gene accessibility and indirectly silencing gene expression. This dynamic interaction between ubiquitous epigenetic machinery and cell type- and sequence-specific repressors enables precise regulation of gene expression, which is crucial for the normal development of diverse cell types and thus essential for life. In the following sections, we discuss selected examples of TFs known to act as repressors of alternative fate programs in tissues derived from the three somatic germ layers and the germline, emphasizing their roles in development (Table EV1). While these examples are not exhaustive, they illustrate the broad involvement of repressors across cell types and germ layers.

### Repressors in the ectoderm

The ectoderm, the outermost germ layer in embryonic development, is crucial as it differentiates into a diverse array of structures. These include the epidermis, hair, nails, and tissues that form the nervous system and sensory organs. Notably, neural crest cells derived from the ectoderm exhibit remarkable versatility, differentiating into various structures such as craniofacial cartilage and bone, peripheral and enteric neurons, glial cells, melanocytes, and even endocrine cells within the adrenal gland. Furthermore, the ectoderm plays a key role in neural tube development, which is the precursor to the central nervous system comprising the brain and spinal cord.

Neurogenesis requires the activation of neuronal gene programs, which must be silenced in non-neuronal cells. The RE-1 Silencing Transcription factor (REST), also known as the neuron-restrictive silencer factor (NRSF), is a classic example of a repressive factor involved in these fate decisions (Schoenherr and Anderson, 1995). REST, a member of the Krüppel-type zinc finger TFs, is ubiquitously expressed. It functions as a negative regulator of neuron-specific gene transcription in non-neuronal cells and plays a critical role in neural differentiation and preservation of the neural phenotype. Despite its broad expression, REST functions in a highly specific manner in vivo. For instance, during astrocytic differentiation of neural progenitor cells, REST is up-regulated and sustained by Bone Morphogenetic Protein (BMP) signal activation, leading to repression of non-astrocyte genes including inhibition of the alternative neuronal identity (Kohyama et al, 2010). Similarly, REST represses terminal maturation genes specifically in zebrafish facial branchiomotor neurons, allowing these neurons to migrate to their target sites (Love and Prince, 2015). While it is known that REST binds to a 21-bp RE1 sites in regulatory regions of its target genes and interacts with subunits of many chromatin modifying enzymes, like CoREST and mSin3 co-repressor complexes, the Switch/Sucrose Non-Fermentable (SWI/SNF) complex, and the Polycomb complex (Andrés et al, 1999; Battaglioli et al, 2002; Dietrich et al, 2012; Grimes et al, 2000), it is still unclear how REST performs its function with such high precision in distinct cell types. An analysis of the REST protein interactome identified amongst others Tripartite motif-containing 28 (TRIM28), also known as KRAB-associated protein 1 (KAP1) (Lee et al, 2016). Intriguingly, TRIM28 has been shown to assist one of the master repressors of the testicular pathway in ovaries, FOXL2, in executing its function via its SUMO-E3-ligase activity (Rossitto et al, 2022) (see the section “Repressors in gonad development: germline vs soma, male vs female”). Thus, one of the mechanisms that potentially ensures specificity of distinct cell fate repressors could be their interaction with cofactors that fine-tune their activity via post-translational modifications.

Unlike REST, Myelin transcription factor 1 like (MYT1L), a zinc finger TF, is a neuron-specific TF and has recently gained attention due to its role as a repressor of multiple non-neuronal cell fates, with significant implications for neuronal safeguarding. MYT1L actively and persistently represses various somatic lineage programs, excluding the neuronal program (Mall et al, 2017). Notably, MYT1L has been implicated in neuronal disorders, including autism spectrum disorder (ASD). Depletion of MYT1L in human neurons and mice results in neurodevelopmental delays, behavioral phenotypes, and ASD-associated gene expression changes reminiscent of alterations observed in patients (Chen et al, 2021; Kim et al, 2022; Weigel et al, 2023). MYT1L deficiency leads to the upregulation of various non-neuronal genes, including the cardiac sodium channel SCN5A, resulting in behavioral and neuronal hyperactivity. Suppressing selected de-repressed MYT1L targets, either pharmacologically or genetically, can partially reverse these phenotypes, even at later developmental stages. This suggests that the continuous and active repression of MYT1L target genes is vital for proper neuronal function. However, it is unclear why MYT1L remains expressed throughout life. MYT1L is reported to mediate long-term silencing through the recruitment of the HDAC-SIN3B transcriptional co-repressor complex (Mall et al, 2017; Romm et al, 2005). It is plausible that epigenetic modifications alone may not suffice to completely silence unwanted gene expression in neurons. Therefore, the lifelong expression of the repressor MYT1L in virtually all neurons could be crucial for active cell fate maintenance. This raises the question of whether sustained expression of repressors is a fundamental requirement for the long-term and persistent maintenance of cell fate decisions, or if this is a feature limited to a subset of lifelong repressors.

The nervous system is complex and composed of different cell lineages with highly specific functions. One model used to resolve mechanisms involved in cell subtype diversification is the differentiation of progenitors into motor neurons (MNs) in the vertebrate neural tube. This process is orchestrated by the basic helix-loop-helix TF Oligodendrocyte transcription factor 2 (Olig2), primarily recognized for its role in oligodendrocytes (Lu et al, 2002; Zhang et al, 2022). Novitch and colleagues, through fusion experiments of activator and repressor effector domains with the DNA-binding bHLH domains of Olig2, revealed that repression of Olig2 target genes is critical for MN differentiation (Novitch et al, 2001). Olig2 does not operate in isolation, but in concert with the two HD TFs Nkx2.2 and Nkx6.1, to directly and broadly suppress components of the Sonic hedgehog pathway and multiple alternative fates arising from neural progenitors (Briscoe et al, 2000; Nishi et al, 2015). A recent single-cell study demonstrated that Olig2 also directly inhibits the expression of Notch signaling pathway effectors Hes1 and Hes5, orchestrating the spatial and temporal pattern of MN generation (Sagner et al, 2018). Together with previous findings highlighting the critical role of PRC1 in specifying MN identity (Sawai et al, 2022), Olig2 and other repressors might execute their inhibitory function in MN specification via PRC1 recruitment.

In addition to MNs, the differentiation of noradrenergic and cholinergic sympathetic neurons relies on a balance of active cross-repression of alternate fates. The H6 family homeobox 1 (HMX1) TF drives noradrenergic sympathetic neuron differentiation by repressing genes typically expressed in cholinergic sympathetic neurons like *Tlx3* and *Ret*. This repression permits *TrkA* and tyrosine hydroxylase (*Th*) expression. Conversely, the repression of HMX1 in cholinergic neurons permits *Tlx3* expression, preventing TrkA and *Th* expression, which is critical for the noradrenergic fate (Furlan et al, 2013). This negative control of alternative programs also extends to the formation of dorsal spinal cord neurons, where PR Domain Zinc finger Protein 13 (PRDM13) is necessary for the repression of excitatory and ventral neural tube programs (Mona et al, 2017). Interestingly, recruitment of PRDM13 to chromatin is facilitated by multiple activating neural bHLH TFs, allowing PRDM13 to repress gene expression at specific loci. Hence, a delicate equilibrium between gene expression activation by these bHLH factors and repression by PRDM13 finely specifies neuronal subtypes, such that loss of PRDM13 leads to derepression in the dorsal spinal cord of ventral neural subtype drivers, including *Olig1*, *Olig2*, and *Prdm12*, causing imprecision in spinal cord neuron specification (Mona et al, 2017). Similarly, the FEZ Family zinc finger 2 (FEZF2) transcriptional repressor is pivotal in specifying cortical projection neurons by suppressing alternative identities. During the specification of corticothalamic and subcerebral neurons, FEZF2 selectively represses the expression of genes inappropriate for each specific neuronal subtype, and its loss leads to defective subcerebral and corticothalamic neuronal development. FEZF2 represses layer 5 subcerebral neuronal genes in layer 6 corticothalamic neurons by recruiting the transcriptional co-repressor TLE4 (Tsyporin et al, 2021). TLE4, a member of the Groucho/Grg/TLE family of co-repressors, guides Polycomb proteins to chromatin (Patel et al, 2012), suggesting a co-repressor-mediated Polycomb-dependent mechanism in the specification of cortical projection neurons.

The emergence of multiple tissues or organs from a single uniform precursor field is a common phenomenon in metazoan development. A prominent example is the *Drosophila* eye-antennal disc, which ultimately gives rise to nearly all external structures of the adult *Drosophila* head including the compound eyes, ocelli, antennae, maxillary palps, head epidermis, and bristles. By studying the eye-antennal disc, Palliyil and colleagues (2018) found that several selector genes, responsible for determining the fates of both the eye and the antenna, are initially expressed throughout the entire homogeneous precursor field. These genes play a crucial role in controlling the growth of the entire disc. As the disc undergoes compartmentalization into distinct territories, gene regulatory networks (GRNs) in these different territories not only continue to promote growth but also take on additional functions, including promoting specific primary fates and suppressing alternative fates (Palliyil et al, 2018). For example, the retinal determination GRN not only promotes the eye fate, but also suppresses the GRN responsible for directing antennal and head capsule fates (Weasner and Kumar, 2013). The mechanism orchestrating timed restriction of expression and expansion of functions of selector genes, a fundamental requirement in fate allocation within a single field, remains unknown. One potential scenario could involve a mechanism akin to the regulation of Gcm expression in the decision between neuronal and glial cells. In this scenario, competition between Gcm and the PRC1 component, Pc, at the *gcm* regulatory region ensures precise silencing of selector gene expression and, consequently, accurate cell fate determination (Popkova et al, 2012). Another possibility could be localized chromatin condensation at specific locations like promoters, a mechanism mediated in the *Drosophila* female germline by SETDB1 (Smolko et al, 2018).

**Figure 4:**
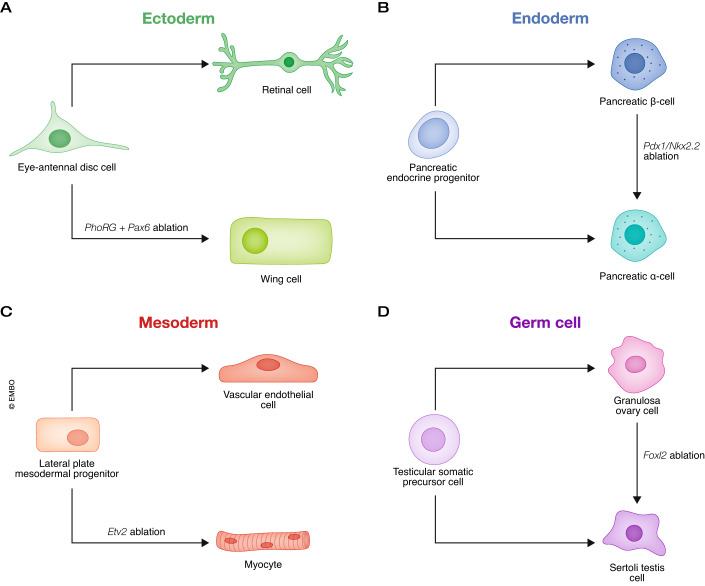
Examples of cell types safeguarded by repressive TFs across all germ layers. (**A**) In *Drosophila*, conditional targeting of the Paired box gene 6 (Pax6) and Pho-repressive complex (PhoRC) PhoRC diverts the developing eye toward a wing fate (Zhu et al, 2018). (**B**) Depletion of Pdx1 or Nkx2.2 in pancreatic beta cells leads to transdifferentiation to alpha cells (Gao et al, 2014; Schaffer et al, 2010). (**C**) Removal of Etv2 in lateral plate mesodermal progenitor cells results in differentiation toward skeletal muscle cells instead of toward vascular endothelial cells (Chestnut et al, 2020). (**D**) Conditional deletion of forkhead box L2 (Foxl2) in adult ovaries causes conversion to Sertoli cells (Rossitto et al, 2022).

The retinal determination GRN has been studied in more detail. Pax6, a Paired-homeobox TF, is a known activator within the eye determination network (Halder et al, 1995). Intriguingly, Pax6 also collaborates with Polycomb to actively suppress alternative fates, playing a pivotal role in maintaining eye fate in both mice and *Drosophila* (Marquardt et al, 2001). Disruption of Polycomb function in the developing eye redirects cells toward a wing fate. When Pax6 and another PcG complex, the Pho-repressive complex (PhoRC), are knocked down simultaneously, the eye-to-wing transformation also occurs (Fig. 4a) (Zhu et al, 2018). Apart from Pax6, members of the SIX family of TFs, namely Sine oculis (So) and Optix, also act as repressors critical for precise eye development. By fusing the Engrailed repressor domain with both So and Optix, thereby mimicking the effects of recruitment by these TFs of their known co-repressor Gro, Anderson and colleagues (2012) revealed that So and Optix trigger ectopic eye formation by functioning as transcriptional repressors in early stages of eye development prevent the expression of non-retinal selector genes (Anderson et al, 2012).

### Repressors in the endoderm

Transcriptional repression is also vital for tissue development and function in the endoderm, another germ layer arising during embryonic development. The endoderm is crucial in the formation of internal organs and tissues in vertebrates and invertebrates. It gives rise to the epithelial linings of various organs, including the digestive tract, respiratory system, liver, pancreas, and glands associated with these systems. In addition, the endoderm contributes to the formation of structures such as the thyroid, thymus, and bladder.

In vertebrate pancreatic development, transcriptional repression is crucial for maintaining tissue identity. The HD TF, NK2 homeobox 2.2 (Nkx2.2), plays a key role in preserving pancreatic β-cell identity. It interacts with a repression complex involving DNMT3a, Grg3, and HDAC1 to repress the homeobox gene *Aristaless* (*Arx*). Disruption of Nkx2.2 function causes ectopic expression of *Arx* in β cells, triggering β- to α-cell transdifferentiation (Fig. 4b). Remarkably, the removal of *Arx* in Nkx2.2 mutant mice reverses this conversion, underscoring the role of Nkx2.2 in maintaining β-cell identity through transcriptional repression of a single alternate fate master regulator (Papizan et al, 2011). Nkx2.2 also regulates oligodendrocyte (OL) differentiation in the nervous system by interacting with the same repression complex via its N-terminal Tinman and C-terminal domains, as shown via electroporation in embryonic chicken (Zhang et al, 2020). Another HD TF with repressive activity, Pancreatic and duodenal homeobox 1 (Pdx1), is essential for both pancreatic development and adult β-cell function. Loss of Pdx1 in β-cells causes *MafB* derepression, severe hyperglycemia, and a significant portion of β-cells transdifferentiating into α-cells (Fig. 4b) (Gao et al, 2014). Therefore, Pdx1 serves as a master regulator of β-cell fate by activating genes crucial for β-cell identity and repressing α-cell genes. In addition, reciprocal repression between the HD TF Nkx6 and the pancreas-specific TF Ptf1a governs the determination of pancreatic progenitor cell fate, specifically between the endocrine and acinar cell lineages. Nkx6 promotes endocrine fate and inhibits acinar fate, while Ptf1a performs the opposite role. This cross-repressive interaction guides progenitors toward their respective lineages, and occurs timepoint-specifically at the multipotent stage of progenitor cells (Schaffer et al, 2010).

Several important alternative fate decisions also need to be taken in the intestine to ensure tissue functionality. For instance, intestinal progenitors differentiate into various secretory cell types, such as enteroendocrine cells (EE) and goblet cells. This decision is controlled by Atonal Related Protein/Achaete-Scute Like (ARP/ASCL) TFs: Neurod1 controls the EE lineage, while Ascl1 and Atoh1 control goblet cell fate. It is so far unknown how these bHLH TFs, which recognize highly similar E-box DNA motifs, orchestrate the acquisition of specific subtype identities. A recent study in zebrafish discovered a 19-amino acid ultra-conserved element (UCE) in Neurod1, absent in other ARP/ASCL proteins, that governs functional divergence. Importantly, the UCE helps mediate goblet cell fate suppression by directing repression of the goblet regulator *gfi1aa*. Removing the UCE from endogenous Neurod1 leads to increased goblet cells and decreased EE cells, mirroring the *neurod1* null mutant phenotype of an altered balance in cell determination. Thus, the UCE plays a critical role in the intestinal activity of Neurod1, potentially influencing cell fate balance via Gfi1 repression in other tissues (Reuter et al, 2022).

In the adult *Drosophila* intestine, the zinc finger TF Klumpfuss (Klu) is crucial in guiding lineage commitment during enterocyte differentiation. Notably, Klu, which is similar to the mammalian tumor-suppressor gene Wilms’ Tumor 1 (WT1), is expressed exclusively in enteroblasts (EBs), the progenitor cells of both enterocytes and enteroendocrine (EE) cells. Decreased Klu levels cause incorrect differentiation of EBs to EE cells instead of enterocytes. Conversely, overexpression of Klu completely inhibits differentiation. Klu influences EE differentiation by repressing genes crucial to Notch signaling and indirectly suppressing the expression of key components of the Achaete-Scute complex (Korzelius et al, 2019). Therefore, by inhibiting the expression of progenitor pathways and alternative fate regulators such as proneural TFs, Klu acts as a safeguard to ensure that EBs remain committed to the enterocyte lineage, and its expression level is a determinant of the proportion of cells that enter each lineage. Previous research has shown an interaction between Klu and the co-repressor Groucho (Gro) in sensory organ precursor cells (Kaspar et al, 2008), suggesting that transcriptional silencing could be mediated by the recruitment of the Polycomb complex to specific sites via Klu-Gro interactions.

### Repressors in the mesoderm

The mesoderm develops into muscle, blood, and related structures, as well as kidney tubules in vertebrates. Several lineage-specific characteristics within these tissues are maintained by adult stem cells. For instance, multipotent hematopoietic stem cells give rise to different blood cell types, including myeloid and lymphoid lineages. These hematopoietic stem cells are transcriptionally primed for differentiation into diverse blood-related lineages. To understand how multipotent cells follow a specific developmental path, Laslo et al investigated the differentiation of macrophages and neutrophils from common myeloid progenitors (Laslo et al, 2006). The study showed that Early growth response protein 1 (Egr-1) and Egr-2, two zinc finger TFs, redundantly govern macrophage fate by activating macrophage-specific genes while suppressing genes associated with the neutrophil lineage. Egr-1 and Egr-2 counteract the activity of Gfi-1, a regulator that determines neutrophil cell fate by repressing genes associated with the macrophage lineage. This counterbalancing repression circuit regulates the balance of differentiation to either macrophages or neutrophils, despite both lineages sharing the same lineage determinants, PU.1 and C/EBPα (Laslo et al, 2006).

PU.1, an ETS-family TF, plays a multifaceted role by promoting myeloid-lymphoid programs and acting as a recognized antagonist of terminal erythroid differentiation. To investigate the specific function of PU.1 within erythroid progenitor cells, Wontakal and colleagues analyzed chromatin interactions involving PU.1 and other essential erythroid-promoting TFs—GATA-1, Stem cell Leukemia (SCL), Krüppel-like Factor 1 (Klf1)—during erythroid cell development. As expected, the study reported that the core erythroid transcriptional network comprising GATA-1, SCL, and Klf1 regulates a substantial repertoire of over 300 erythroid-specific genes (Wontakal et al, 2012). The study also found that PU.1 directly binds and represses numerous genes within the core network, in addition to silencing the expression of GATA-1, SCL, and Klf1 themselves. This observation highlights a dual mechanism wherein PU.1 exerts repression on both the erythroid-promoting master regulators and their downstream targets (Chou et al, 2009; Wontakal et al, 2012), thereby generating a synergistic and robust mechanism crucial for precise lineage specification.

Lymphoid progenitors can generate natural killer cells and small lymphocytes, primarily T and B cells. T-cell identity is defined by several TFs including T-cell factor 1 (TCF-1) and GATA-3. While TCF-1 induces T cell specific-genes (Weber et al, 2011), GATA-3 plays a dual role by promoting and upregulating the core transcriptional program specific to T cells, including the activation of Notch1, and simultaneously suppressing alternate fates by repressing natural killer cell and B cell-specific genes (García-Ojeda et al, 2013; Van de Walle et al, 2016). These findings highlight GATA-3 as an example of a factor that establishes a feedback loop, exerting positive and negative regulation to induce a distinct cellular fate. Conversely, the fate of B cells is facilitated by EBF1. Banerjee et al showed that EBF1 not only represses GATA-3 transcription in B cells but also facilitates the establishment of repressive histone modifications (H3K27me3) at the GATA-3 gene locus in B cells (Banerjee et al, 2013). This indicates not only that the activation of alternative fate genes can be repressed by a lineage-specific repressor, but also that long-term memory of cell fate decisions can be established through the actions of such safeguard TFs. Thus, the differential fates of T and B cells are governed by intricate regulatory networks involving safeguard TFs such as GATA-3 and EBF1, which orchestrate a delicate balance of positive and negative feedback loops to guide and consolidate specific cell fate decisions and suppress alternate cell fates.

The mesoderm also gives rise to the somatic musculature, which comprises syncytial myocytes formed through myoblast fusion. In invertebrates, mesodermal cells generate the somatic, cardiac, visceral, and fat body (analogous to the liver), while in vertebrates, somatic muscle progenitors are believed to originate from somites. Conversely, the lateral plate mesoderm contributes to the development of vascular endothelial cells, hematopoietic lineage, and cardiomyocytes. A study by Chestnut et al on the expression requirements of the ETS TF Etv2/Etsrp during vascular development in zebrafish revealed an unexpected finding. The loss of Etv2/Etsrp function resulted in a shift in cellular fate differentiation from the vascular lineage to the somatic muscle lineage (Fig. 4c) (Chestnut et al, 2020). These findings strongly suggest that Etv2/Etsrp functions as a suppressor of the alternative fates within the multipotent mesodermal lineage to stabilize vascular development. Moreover, the study demonstrates that the lateral plate mesoderm can generate somatic progenitors, highlighting the remarkable plasticity and multipotency of these cells within the mesodermal context.

The general mechanism of muscle development shows similarities in both invertebrates and vertebrates. It involves a muscle founder cell that carries crucial information regarding the future shape, attachment, and orientation of the muscle. Fusion-competent myoblasts then fuse with the founder cell, ultimately forming functional muscle fibers. Extensive research in Drosophila has shown how distinct myoblast fates are established through positive cues. Numerous identity genes have been identified that play defining roles in determining the fate of different founder cells. An illustrative example of founder cell identity involves the transcriptional influence of two Nk-like homeobox TFs, Ladybird (Lb) and Slouch (Slou). Lb determines the identity of the segment boundary muscle (SBM), while Slou defines the ventral acute 2 muscle (VA2) (Knirr et al, 1999). Loss of Slou function leads to the development of two SBMs and the absence of VA2, suggesting that Slou suppresses Lb in SBM to establish the VA2 identity (Knirr et al, 1999). Lame duck (Lmd) and Tramtrack (Ttk), two zinc finger TFs, also regulate the fate of fusion-competent myoblasts (FCMs). Lmd activates a FCM-specific transcriptional program while Ttk directly represses the transcriptional program for the alternative lineage, the muscle founder cell (Ciglar et al, 2014). This exemplifies the idea that a minimum of two TFs exerting opposing influences can effectively regulate cell fate decisions. One TF functions as an activator, facilitating the expression of lineage-specific programs, while the other TF acts as a repressor, inhibiting alternative cellular outcomes.

The role of TFs in repressing alternative fates to ensure tissue differentiation has been studied in another context related to the mesoderm. Domsch et al analyzed the Drosophila Hox TF Ultrabithorax (Ubx) in somatic muscles and investigated how this TF, which is expressed in multiple cell and tissue types, controls the development of mesodermal cells in a highly specific manner. Their analysis revealed that mesoderm-specific Ubx depletion resulted in the de-repression of genes typically expressed in other alternative lineages. Intriguingly, Ubx silences alternative fate genes by localizing the Polycomb Group protein Pleiohomeotic (Pho) at Ubx-targeted genomic regions, thereby stabilizing repressive H3K27me3 chromatin marks in a mesoderm-dependent manner (Domsch et al, 2019). This demonstrates that Ubx enhances the determination of cellular lineages by inhibiting the latent multipotency encoded in the genome through its interaction with Pho. Two key insights can be derived from this study. Firstly, TFs with generic expression patterns can recruit chromatin remodelers with repressive properties to precise genomic loci, enabling the selective suppression of highly specific genes that govern alternative fate development. Such specificity, however, requires interactions with more functionally-restricted factors, which so far remain unknown. Secondly, considering that Hox TFs are expressed throughout an organism’s lifespan (similar to MYT1L in the vertebrate nervous system, see the section “Repressors in the ectoderm”), the Hox-PcG-mediated repression of genes associated with alternative fates could impose a barrier to prevent cell fate instability or even trans-differentiation in adult cells. This notion is particularly interesting for cell reprogramming or transdifferentiation.

### Repressors in gonad development: germline vs soma, male vs female

Two critical decisions are made during gonad development. The first decision involves ensuring that specified germ cells maintain their germline trajectory and do not adopt a somatic fate. The second decision arises once the gonads have formed, determining whether they will differentiate into testes or ovaries. Despite the extensive knowledge about the epigenetic regulation of these processes (Cheng et al, 2022; Strome and Updike, 2015), our understanding of the TFs or GRNs that orchestrate the repression of alternative fates remains limited.

Sybirna et al, 2020 examined the role of PRDM14, a key transcriptional regulator of mouse primordial germ cells (PGCs), in human PGCs (hPGCs) derived in vitro from human embryonic stem cells. Analysis of target gene expression in PRDM14-depleted cells revealed cooperation with two additional TFs, TFAP2C and Blimp1 (also known as PRDM1), to directly repress Wnt signaling and somatic genes, particularly those associated with heart and brain development, to ensure proper PGC differentiation. Interestingly, it has been previously reported that Blimp1 represses somatic genes in mouse PGC-like cells by selectively recruiting HDAC3 to these target genes (Mochizuki et al, 2018), suggesting that the selective recruitment of epigenetic regulators by the combinatorial action of TFs enables the silencing of somatic genes in the germline. This study raises two related questions: why do PRDM14, TFAP2C, and Blimp1 predominantly repress heart and brain-related genes; and whether there are additional TFs that are active in the germline to repress other somatic alternatives. The latter seems to be true, as demonstrated in the Drosophila male germline. In this lineage, the spermatocyte-specific zinc finger protein, Kumgang (Kmg), cooperates with the chromatin remodeler dMi-2 to block the action of a promiscuous activator on cryptic promoters to prevent transcription of genes normally expressed only in the somatic lineage. Thus, faithful gene activation can rely on repressors that block normally cryptic promoters, which could become active when new regions of the genome open for transcription during terminal differentiation (Kim et al, 2017).

Gonads must also differentiate into a female or male fate. In mammals, gonadal sex is determined by the presence or absence of the Y-linked TF Sry which induces expression of the HMG-box TF Sox9 and directs Sertoli cell differentiation in XY bipotential gonads. In XX gonads lacking Sry, the WNT4-β-catenin signaling pathway regulates expression of the forkhead TF FOXL2 in somatic cells, which differentiate into granulosa cells in developing ovarian follicles at later developmental stages. Thus, Sox9 and FOXL2 are two key TFs active in developing bipotential gonads that determine their sex. To understand FOXL2 function during gonad differentiation, Nicol et al profiled chromatin occupancy of FOXL2 in fetal ovaries, which they correlated with transcriptional changes upon Foxl2 gain- and loss-of-function (Nicol et al, 2018). This analysis revealed that FOXL2 likely directly represses the pro-testis factor Sox9 via a long-distance regulatory element as well as the cell-cycle repressor Cdkn1b in XX gonads, which could explain the limited growth of ovaries during sex determination. Comparison the genome-wide occupancy of FOXL2 in the fetal ovary with that of the testis-determining factor Sox9 in the fetal testis, indicated significant overlaps between the two (Nicol et al, 2018), suggesting antagonistic or redundant signals between FOXL2 and Sox9 at the chromatin level. Gonadal sexual fate in mammals must be actively maintained in adulthood, a process that is also regulated by FOXL2. Interestingly, a recent study showed that FOXL2 protects granulosa cells from trans-differentiating into Sertoli cells by recruiting TRIM28 to repress the Sertoli cell pathway via the SUMO-E3-ligase activity of TRIM28 (Fig. 4d) (Rossitto et al, 2022). Thus, TRIM28 acts in concert with FOXL2 to protect the adult ovary from inducing testis-specific genes, possibly by sumoylating FOXL2 and other chromatin-binding proteins, thereby promoting their stabilization and long-term silencing of testis genes in the adult ovary.

## Transcriptional repression during reprogramming

Cell fate plasticity allows differentiated cells to undergo transdifferentiation and reprogramming, enabling them to change into other cell types or even pluripotent states. This process involves the transcriptional rewiring of cellular identity and can occur naturally or be induced artificially (Aydin and Mazzoni, 2019). Transdifferentiation and reprogramming can be divided into three intertwined phases: initiation, maturation, and stabilization (David and Polo, 2014). During the initiation phase, cells undergo the induction of a new cell identity program and the repression of donor cell specific genes. The primary driver of reprogramming is often target gene activation induced by master TFs with pioneer function (Foo et al, 2014; Harrison et al, 2011; Iwafuchi-Doi et al, 2016; Lee et al, 2013; Nien et al, 2011; Pan and Schultz, 2011), which can access and open closed chromatin to activate genes (Soufi et al, 2015; Wapinski et al, 2013). Consequently, strategies that increase overall chromatin accessibility and activate gene expression are widely used to promote cell fate conversions. However, epigenetic barriers, residual expression of donor cell genes, or even induction of alternate genes can delay or hinder the induction of a desired cell identity and consequently active repression has recently emerged as critical mechanism during reprogramming

### Partial reprogramming by global derepression

The first reported chemical-induced cell fate reprogramming involved the use of 5-azacytidine, a DNA methyltransferase inhibitor, to induce the expression of muscle-specific genes in fibroblasts (Taylor and Jones, 1979). Following this, Harold Weintraub and colleagues (Davis et al, 1987) identified Myod1 as the master regulator TF responsible for this conversion. Targeted DNA demethylation of the Myod1 enhancer using a dCas9-Tet1 fusion construct has also been shown to further enhance fibroblast-to-muscle cell reprogramming by activating Myod1 expression (Liu et al, 2016). Myod1 can convert certain cell types, like fibroblasts and adipocytes, into muscle cells, but it has limitations in fully converting other cell types. For instance, melanoma and neuroblastoma cells gain muscle marker expression upon Myod1 expression but retain features of their donor cell types, such as pigment granules and axon-like projections, indicating that donor cell identity and epigenetic barriers influence cell fate plasticity during reprogramming (Weintraub et al, 1989). Similarly, the neuronal reprogramming factor Ascl1 can convert fibroblasts toward neurons (Chanda et al, 2014), but is unable to convert keratinocytes (Wapinski et al, 2013), again illustrating the influence of donor cell identity and epigenetic barriers.

Histone deacetylase (HDAC) inhibitors, which prevent histone deacetylation-mediated chromatin compaction, can also enhance cell fate conversion. For instance, they enhance the conversion of cardiac rat fibroblasts to cardiomyocytes and mouse embryonic fibroblasts toward neural progenitor cells (NPCs) (Cheng et al, 2014; Singh et al, 2020). However, uncontrolled alteration of global chromatin accessibility can impair cell fate and function. In TF-mediated conversion of iPSCs to neurons, the HDAC inhibitor valproic acid (VPA) led to the upregulation of genes blocking neuronal differentiation, such as ID3, impairing neuronal morphology and function in the early phases of cell fate conversion (Chanda et al, 2019). This can explain the teratogenic effects of VPA in fetal valproate syndrome, which includes severe cognitive defects, and highlights how uncontrolled global relief of repressive chromatin can have unwanted or deleterious effects.

### Enhanced reprogramming by specific repression

Precise repression of donor cell or alternative gene expression programs is pivotal in preventing “cell-fate confusion” particularly during cell fate maturation and stabilization. This targeted repression is illustrated by the role of microRNAs (miRNAs) in cell fate determination. Cell type-specific miRNAs play an important role in enhancing cell fate conversions by directly repressing target mRNAs encoding TFs of competing cell fates. For example, miR-122 silences the fibroblast TF Snail1, promoting conversion of fibroblasts to cardiomyocytes (Jayawardena et al, 2012; Muraoka et al, 2014). Likewise, miR-9/9∗ and miR-124 can convert fibroblasts to neurons, partly by suppressing the neuronal gene repressor REST (Lee et al, 2018; Yoo et al, 2011). These examples underscore the critical role of miRNA-mediated inhibition of competing fates.

Interestingly, the reprogramming factors themselves can also mediate this process. During the reprogramming of microglia to neurons, the proneural TF NeuroD1 induces the repressors Scrt1 and Meis2, which subsequently mediate the suppression of microglial programs by silencing TFs that drive immune cell development and maintenance, including Lyl1 and Mafb (Matsuda et al, 2019). Since depletion of Lyl1 and Mafb causes destabilization of microglia identity, silencing of donor cell-specific TFs appears to be a crucial step triggered indirectly by the activation of donor cell fate-specific repressors. Furthermore, chemical inhibition or genetic downregulation of the H3K79 histone methyltransferase DOT1L, which has been reported to counteract gene repression, accelerates reprogramming of fibroblasts toward pluripotency (Onder et al, 2012). In this context, DOT1L inhibition decreases H3K79me2 at several fibroblast master regulators, such as SNAI1&2 and ZEB1&2, consequently repressing their expression.

Computational methods have also been employed to identify TFs that maintain a specific fate and counteract reprogramming. CellNet, a computational method to identify TFs that would maintain a specific fate and hereby counteract reprogramming, predicted that Pou2 associating factor 1 (Pou2af1) and Early B cell factor 1 (Ebf1) could counteract B cell to macrophage conversion. Depletion of both factors indeed enhanced reprogramming toward the macrophage fate, reinforcing the notion that the active silencing of master regulators of the donor cell fate significantly enhances cell fate conversions (Morris et al, 2014).

### Stable reprogramming by donor and alternate fate repression

Residual donor cell identity can impede reprogramming, whether that is performed by nuclear transfer (NT) or direct reprogramming, and its effects can be observed in the resulting cells. For instance, during NT of a neurula-stage endoderm cell into an enucleated Xenopus cell, an epigenetic ON-memory marked by the histone modification H3K4me3 caused increased expression of endoderm genes in ectoderm cells of the NT embryo. Overexpression of the repressive H3K4-specific demethylase Kdm5b decreased the donor signature expression and increased successful development following NT (Hörmanseder et al, 2017). Similarly, MNs directly reprogrammed from mouse embryonic fibroblasts using TFs retained anterior-posterior positional identity encoded by specific Hox genes expressed in the starting cells (Ichida et al, 2018). However, MNs programmed from iPSCs acquired generic MN identity and could be regionalized using morphogen cues like the caudalizing signal retinoic acid (Mazzoni et al, 2013). This indicates that cell identity induction can be separated into two dimensions: the initial induction of an overall cell or lineage identity and the fine-tuned establishment of features like regional or subtype identity, both of which require orchestrated induction and repression of cell-type-specific GRNs.

Loss of repressors can also destabilize established cell fates and cause disease. For instance, the repressor retinoblastoma (Rb) is a classic tumor suppressor that prevents malignant transformation by repressing several tumor-promoting processes including the cell cycle (Burkhart and Sage, 2008). Unexpectedly, Rb inactivation promotes the reprogramming of differentiated cells toward pluripotency not by enhancing cell proliferation, but by de-repressing pluripotency genes, including the direct Rb target genes Sox2 and Oct4. Thus, impaired repression of pluripotency master TFs upon Rb loss promotes dedifferentiation that can contribute to tumor formation (Kareta et al, 2015). These examples emphasize that the cell of origin strongly influences the success of cell fate conversion and that silencing the starting cell fate is a key barrier in reprogramming experiments. Furthermore, active repression by specific TFs can regulate distinct stages of cell fate reprogramming.

The importance of active repression in cell fate conversions has been further supported by studying the trajectories of mouse embryonic fibroblasts (MEFs) during reprogramming to induced neurons (iNs) using single-cell transcriptomics. These experiments revealed that reprogramming factor overexpression produced either successfully reprogrammed iN cells, a putative ‘dead-end’ fibroblast state, or, unexpectedly, myocyte-like cells (Treutlein et al, 2016). Similarly, trajectories of single cells during the conversion of MEFs to induced endoderm progenitors (iEPs) revealed that the cells follow either a successful path or an alternate path to a ‘dead-end’ state characterized by re-expression of fibroblast genes (Biddy et al, 2018). Such “cell-fate confusion” observed during reprogramming may arise from the promiscuity of TF binding, as seen with the pro-neuronal TF Ascl1 and the muscle-inducer Myod1, both of which possess conserved bHLH domains which can bind to similar E-Box DNA motifs (Lee et al, 2020).

**Figure 5:**
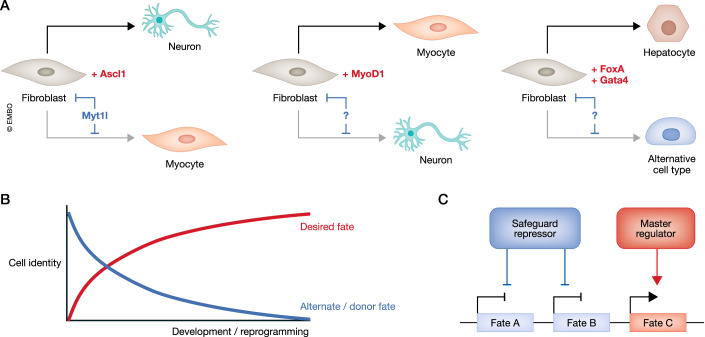
Model of cell-type specific safeguard repressors that facilitate reprogramming and development by active repression of alternative cell fates. (**A**) Cells such as fibroblasts can be reprogrammed to other cell types by expression of master regulators. Notable examples include the pioneer factors Ascl1, MyoD1, and FoxA/Gata4, which drive reprogramming to neurons, myocytes, and hepatocytes, respectively. During the conversion of fibroblasts to neurons, an alternative muscle fate can be induced by the promiscuous activity of the neuronal master regulator Ascl1 (Lee et al, 2020). Repression of muscle-related gene expression as well as fibroblast-specific donor cell genes by the neuron-specific safeguard repressor Myt1l prevents unwanted gene expression and increases the efficiency and fidelity of induced neuronal reprogramming (Mall et al, 2017). Similarly, MyoD1-induced myocyte reprogramming also generates an alternative neuronal fate, which could be repressed by an as-yet unknown factor. Finally, FoxA+Gata4 reprogramming to hepatocytes may induce alternative cell identities which themselves also need to be repressed. (**B**) In general, efficient reprogramming and development requires not only an increase in expression of desired fate genes, but silencing of genes associated with the donor fate and/or alternative identities. (**C**) In this model, cell-type specific safeguard repressors that silence alternate cell identities might work hand in hand with cell-type specific master regulators to faithfully induce and maintain a specific cell fate.

However, direct and indirect mechanisms ensure that the transcriptional outputs of both TFs are substantially different. Direct cofactors like the HD TF PBX interact with Myod1 to fine-tune binding toward muscle-enriched genes. Conversely, indirect cofactors prevent unwanted induction of muscle genes by the ‘on-target’ pioneer TF Ascl1 without altering its DNA binding (Wapinski et al, 2013). In this case, the neuron-specific repressor Myt1l can corral the promiscuity of Ascl1 by directly repressing many non-neuronal GRNs, including fibroblast and muscle-specific genes (Fig. 5a) (Mall et al, 2017). Hence, unlike REST, Myt1l acts as a repressive safeguard of neuronal cell fate by suppressing many non-neuronal master regulators and downstream genes.

Besides Ascl1 and Myod1, several other pioneer factors, including the hepatocyte inducers FoxA and Gata4 (Fig. 5a) (Cirillo et al, 2002), also act as master regulators of cell identity during development and reprogramming based on their ability to bind and initiate opening of silent chromatin regions (recently reviewed in (Barral and Zaret, 2023)). Systematically studying the binding of additional pioneer factors using methods such as chromatin immunoprecipitation sequencing (ChIP-seq), cleavage under targets and release using nuclease (CUT&RUN), cleavage under targets and tagmentation (CUT&Tag) (Park, 2009; Skene and Henikoff, 2017; Kaya-Okur et al, 2019), combined with studying the chromatin accessibility before and after induction of reprogramming via DNase or micrococcal nuclease (MNase) treatment or Assay for Transposase-Accessible Chromatin (ATAC) followed by sequencing, will help to understand whether pioneer factors in general can render unwanted genes accessible (Johnson et al, 2006). For instance, like Ascl1, Myod1 may require additional repressor activity for high-fidelity reprogramming (Lee et al, 2020). In this case, regulation by safeguard repressors could be key in reprogramming cells with desired identities for disease modeling and biomedical applications. Future studies will be needed to uncover similar cell-type-specific safeguard repressors in various lineages that actively repress donor and unwanted fates, ensuring faithful cell fate conversions for applications in disease modeling and regenerative medicine (Fig. 5a).

## Conclusions

While activation is undoubtedly vital to regulate cell fate, a growing body of evidence suggests that active repression plays a critical role in regulating cell identity. Repression acts as a constraint, narrowing down the available options upon which activators can exert their influence. The examples highlighted in this Review show that a delicate balance between activation and repression is fundamental for achieving precise and controlled cell fate decisions during development. As authors, we do not ask which process, repression or activation, holds greater significance. Instead, the data suggest that a combination of active repression and activation is required to ensure faithful cell fate induction and maintenance (Fig. 5b). However, we do believe that repression is understudied relative to its importance. The examples presented here in different cell types from all germ layers across different species demonstrate the ubiquity of repression in regulating cell fate.

It is evident that there exist several layers of magnitude in alternative fate repression, ranging from major decisions such as germ layer choice, to fine-grained cell subtype distinctions within the same tissue, or to small changes in cell states. In this layer model, cells could progressively suppress alternative fates during development, ultimately resulting in highly specialized cell types aided by repressor-guided canalization (Fig. 1). Mechanistically, we speculate that the magnitude of fate guidance by repression between major conversions may involve substantial chromatin restructuring, such as the formation of liquid-liquid phase-separated sub-nuclear structures like Polycomb bodies (Kim and Kingston, 2020), while minor decisions may only involve silencing specific master regulatory genes. Novel epigenome editing tools such as CRISPR/Cas9 will help to address this experimentally (Breunig et al, 2021; Wang et al, 2016).

For repressors to achieve specificity in how they canalize cell fate, the ubiquitously-expressed and non-specific silencing machinery must collaborate with sequence-specific TFs. These TFs can be expressed in a cell-type-specific manner and bind to cis-regulatory elements of genes that are not native to that cell type, thereby suppressing their expression. This form of specificity would allow a TF to act as a safeguard repressor in a specific cell type to silence many unwanted gene programs simultaneously using ubiquitously available co-repressors (Fig. 5c). Indeed, some safeguard repressors have been identified in various cell lineages and species, such as Myt1l in mouse neurons and Kmg in the fly germline (Table EV1) (Kim et al, 2017; Mall et al, 2017). The discovery of safeguard repressors could be further facilitated through bioinformatic prediction. Candidates for safeguard repressors could be identified using cell type-specific GRN models derived from data on gene expression, chromatin accessibility in single cells, TF binding, and the occurrence of known repressor domains within TFs. Integrating these data sources can enhance our ability to identify and understand the function of safeguard repressors in various cell types (Janssens et al, 2022; Klaus et al, 2023).

Though more safeguard repressors may exist, many TFs have dual functions as repressors and activators, often in a cell type-specific and context-dependent manner, regulating desired genes while suppressing unwanted programs (Fixsen et al, 2023; Revilla-i-Domingo et al, 2012). Importantly, most of the TFs involved in alternative fate repression are expressed in multiple lineages (Table EV1) and perform their function in a highly context-dependent manner. Thus, it seems likely that many cell fate decisions function through the dynamic assembly of repressive complexes comprising multiple TFs, cofactors, and epigenetic factors. These complexes act in a context- and stage-specific manner to establish barriers that guide activating transcriptional complexes within cells toward their final destination (Figs. 2 and 3). Identifying components of such dynamic complexes will likely require targeted approaches to capture temporary protein-protein interactions in a cell-specific manner. Techniques like TurboID or split-TurboID, which are enzyme-catalyzed proximity labeling methods for one (TurboID) or two (split-TurboID) selected proteins, could offer insights by revealing short-lived interactions of specific proteins in living cells (Branon et al, 2018; Cho et al, 2020). With both methods, the high labeling efficiency and speed of the labeling enzyme would help provide cell type-specific and temporal information about dynamic repressor protein interactions in their native cellular context, providing a deeper understanding of the machinery and mechanisms involved in alternative fate repression.

Systematic studies investigating the roles of specific TFs and their co-repressors at individual enhancers/silencers and linked target genes will also help unravel the regulatory logic and molecular mechanisms that determine a target’s sensitivity or resistance to repression (Jacobs et al, 2023). It is conceivable that a TF can be repurposed to mediate repression in one lineage and activation in another, depending on changing direct interaction partners, post-translational modifications, or the availability of additional TFs binding to nearby regulatory regions. This would enable cell type specification based on limited numbers of TFs and cis-regulatory DNA-binding motifs at cell type specific genes. Engineering artificial cell fate regulators fusing the DNA-binding domains to constitutive activator (i.e., VP64) or repressor (i.e., Engrailed) domains, will help to address this hypothesis and reveal the primary functions of TFs in cell fate specification and maintenance. Gain- or loss-of-function screens across all TFs, coupled with multi-omics readouts, can also identify novel repressors safeguarding cell identities (Ng et al, 2021).

Elucidating alternative fate repression dynamics and mechanisms will also be critical for understanding human diseases. The loss of lifelong-expressed TFs which cement cell fate may contribute to cancer and age-related degeneration associated with increased plasticity (Hanahan, 2022; Traxler et al, 2023). Exploring the intricacies of these mechanisms will provide profound insights into cellular development and diseases, but will also require exploring multiple levels of repression and recognizing the presence of undesirable fate opportunities within cells. By unraveling these mechanisms, we will gain profound insights into cellular development and disease, while facilitating the progress of fields such as regenerative medicine and tissue engineering.

### Supplementary information

Table EV1
